# Contrasting patterns of phylogeographic relationships in sympatric sister species of ironclad beetles (Zopheridae: *Phloeodes *spp.) in California's Transverse Ranges

**DOI:** 10.1186/1471-2148-10-195

**Published:** 2010-06-24

**Authors:** Maxi Polihronakis, Michael S Caterino

**Affiliations:** 1 Department of Invertebrate Zoology, Santa Barbara Museum of Natural History, 2559 Puesta del Sol Rd, Santa Barbara, CA 93105 USA

## Abstract

**Background:**

Comparative phylogeography of sympatric sibling species provides an opportunity to isolate the effects of geography and demographics on the evolutionary history of two lineages over the same, known time scale. In the current study, we investigated the phylogeographic structure of two zopherid beetle species, *Phloeodes diabolicus *and *P. plicatus*, where their ranges overlap in California's Transverse Ranges.

**Results:**

Although *P. diabolicus *and *P. plicatus *share similar habitats with largely overlapping distributions, the results of this study revealed different evolutionary histories for each species since divergence from their most recent common ancestor. In general, *P. plicatus *had higher genetic diversity, and more among population isolation than *P. diabolicus*. The mismatch distributions indicated that one major difference between the two species was the timing of population expansion. This result was consistent with genetic patterns revealed by the Φ_st _values and genetic diversity. Lastly, there were no parallel genetic breaks at similar geographic barriers between the species.

**Conclusions:**

Our data revealed that differential demographics rather than geography were responsible for the genetic patterns of the two species.

## Background

Phylogeographic studies seek to understand how current and historical habitat dynamics influence lineage divergence and species diversity patterns. Integrating data from multiple species in a specific region makes it possible to assess trends in the data that help identify large-scale phenomena that contribute to regional species diversity patterns [1,2]. However, comparing phylogeographic structure across many disparate species introduces many spatio-temporal and life history variables that are difficult to accommodate. For example, whether multiple species exhibit parallel genetic patterns is dependent on factors such as dispersal ability and the timing of population divergence [3]. One way to address these discrepancies and isolate the factors affecting current distribution patterns is to study sympatric sibling species [4]. Comparing sympatric sister species provides a means to directly compare the evolutionary history of two lineages on a known relative time scale. Such comparisons provide a basis to tease apart the role of general phenomena such as phylogeographic barriers versus species-specific natural history traits and demographic variables in extant patterns of diversity.

The current study uses multi-locus sequence data to reconstruct the evolutionary history of two species of ironclad beetle (family Zopheridae), *Phloeodes diabolicus *(LeConte) and *Phloeodes plicatus *(LeConte). Due to recent changes in the higher level classification of the family involving the focal taxa of the current study, we follow the work of Foley and Ivie [5] and recognize the distinction of the genus *Phloeodes *from *Nosoderma *and *Noserus *whose synonymy was proposed by García-París et al. [6]. The dynamic taxonomic history of beetles in the family Zopheridae in part reflects the variability of the morphological characters used by various authors to diagnose species, such as the degree of elytral sculpturing and texture. In 1907, the variation observed in this group led Casey [7] to describe six species that have since been synonymized with *Phloeodes diabolicus *[5,8], and four species that have been synonymized with *Phloeodes plicatus *[5]. This high level of morphological variation in combination with an unstable taxonomic history suggests an interesting evolutionary story for these beetles that we investigate using genetic data.

*Phloeodes diabolicus *and *P. plicatus *are two of three species in this genus and are hypothesized to be sister taxa relative to the third species, *P. venustus*, found in Central America [5]. Both species are broadly distributed in the California Floristic Province, are flightless, and can be found under bark feeding on fungus in dead hardwood and coniferous trees. *Phloeodes diabolicus *is found as far north as Mt. Shasta (near the California/Oregon border), and into both the northern and southern Sierra Nevada Mts. On the other hand, the distribution of *P. plicatus *is limited to the coastal ranges of California and the southern Sierra Nevada Mts. Nevertheless, large portions of their range do overlap, and individuals of the two species are often found in microsympatry (sometimes under the bark of the same tree). Thus, the first goal of this study was to determine whether these two lineages are reproductively isolated or if there has been gene flow in the parts of their range where they overlap. We then examined the genetic patterns associated with the broad distribution of *P. diabolicus *in comparison with the relatively restricted distribution of *P. plicatus*. Specifically, we compared historical demography, phylogeographic structure, genetic diversity, and tested whether the species exhibited genetic breaks at similar biogeographic barriers.

## Results

**Figure 1:**
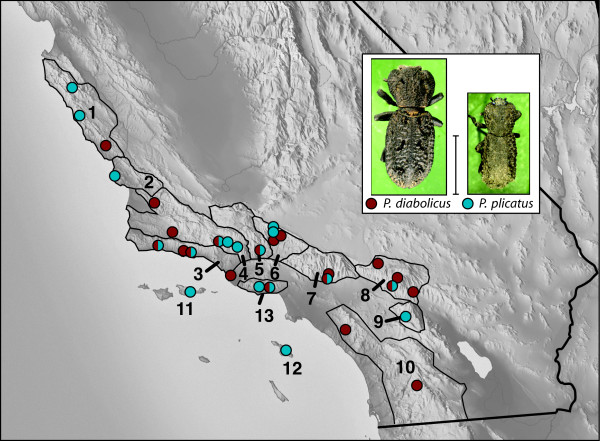
**Map of southern California illustrating collecting localities for *P. diabolicus *and *P. plicatus *specimens**. Some dots may represent two or more collecting localities that are in close proximity (see Additional File 1). Images of *P. diabolicus *and *P. plicatus *are to scale; scale bar ~ 1.0 cm. Regions are numbered as follows: 1. Northern Santa Lucia Mountains; 2. Southern Santa Lucia Mountains; 3. Santa Ynez Mountains; 4. Northwest Transverse Ranges; 5. Central Transverse Ranges; 6. Sierra Pelona; 7. San Gabriel Mountains; 8. San Bernardino Mountains; 9. San Jacinto Mountains; 10. Peninsular Ranges; 11. Northern Channel Islands (Santa Cruz Island); 12. Southern Channel Islands (Santa Catalina Island); and 13. Santa Monica Mts. The Transverse Ranges comprise regions 3-9.

Our sampling effort yielded 53 specimens of *Phloeodes diabolicus*, and 36 specimens of *Phloeodes plicatus *(Figure 1). Amplification of the latter half of COI included 824 base pairs, of which 50 were variable and 25 were parsimony informative in *P. diabolicus*; and 99 variable with 61 parsimony informative in *P. plicatus *(GenBank accessions HM026069-HM026125; Additional File 1). This variation resulted in 26 different *P. diabolicus *haplotypes and 31 different *P. plicatus *haplotypes. For the 762 base pair portion of the CAD gene amplified, there were 18 variable and 7 parsimony informative sites in *P. diabolicus*; and 47 variable and 42 parsimony informative sites in *P. plicatus *(GenBank accessions HM026126-HM026169; Additional File 1). Of the 28 *P. diabolicus *individuals for which CAD was amplified, 12 were heterozygous yielding a total of 16 haplotypes. For *P. plicatus*, 16 of 20 individuals were heterozygous at the CAD locus and yielded a total of 28 haplotypes. Thus, for both genes amplified, *P. plicatus *had smaller sample sizes but more variable sites and more unique haplotypes than *P. diabolicus*.

The gametic phase of heterozygous individuals was inferred using the program Phase v2.1 [9,10]. There were several individuals from both species that had one or two positions that could not be determined with probability >0.95 and so these positions were coded using the IUPAC ambiguity codes. There was no statistically significant evidence for recombination in the CAD gene (p = 0.68) in *P. diabolicus*, but there was in *P. plicatus *(p = 0.00). Because recombination violates the assumption of a bifurcating tree structure inherent to all phylogenetic methods, we interpreted CAD haplotype relationships inferred through network analysis because it allows for reticulation and reflects recombination by the presence of loops [11].

### Phylogenetic and Network Analyses

**Figure 2:**
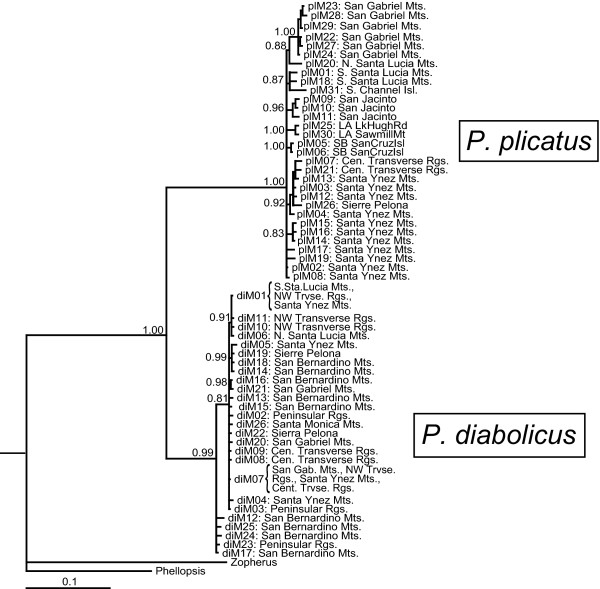
**COI gene tree inferred in MrBayes**. Terminals denote haplotype numbers (corresponding to Figs. 3a, 3b, and Additional File 1) and region collected. Branch lengths with posterior probability <0.80 collapsed.

**Figure 3:**
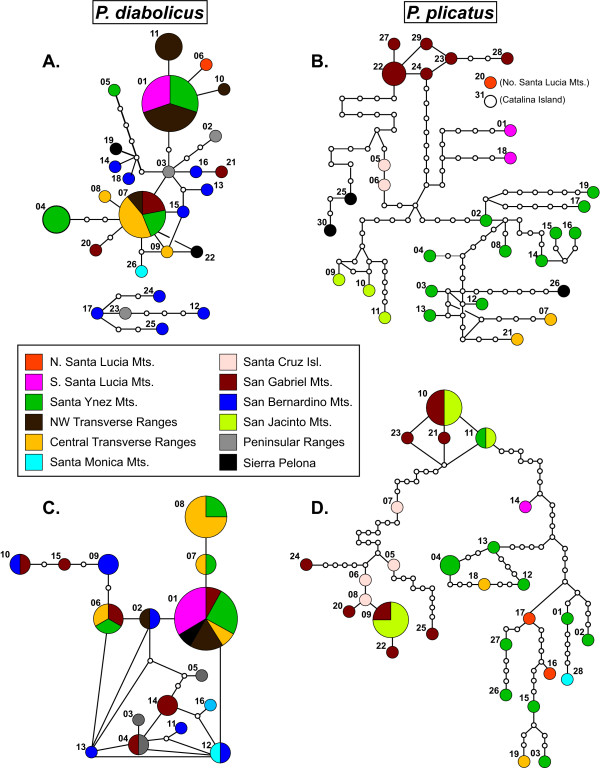
**TCS haplotype networks based on COI and CAD**. Haplotypes color coded by region; size of circle is proportional to haplotype frequency. Numbers next to circle denote haplotype numbers and correspond with localities in Additional File 1. a) *P. diabolicus *COI haplotype network, b) *P. plicatus *COI haplotype network, c) *P. diabolicus *CAD haplotype network, d) *P. plicatus *CAD haplotype network.

The COI gene tree from MrBayes had strong support for the monophyly of both *P. diabolicus *and *P. plicatus *(Figure 2). There was also support for smaller subgroups within each of these lineages, but relationships among these were largely unresolved and resulted in large polytomies. Within the *P. diabolicus *lineage, TCS resolved two separate haplotype networks, a small one comprising five haplotypes from relatively arid localities in the southern Peninsular Range and San Bernardino Mts., and the other with 21 haplotypes from the northern Peninsular and Transverse Ranges (Figure 3a). These two networks overlapped in the eastern Transverse Ranges (San Bernardino Mts.) as a result of one haplotype from Willow Creek (diM12) in the smaller network while all other haplotypes from Willow Creek (diM13-diM16) were in the larger network. Within the larger haplotype group, there were three small subgroups that showed weak geographic congruence. The first cluster included the most frequent *P. diabolicus *haplotype (diM01), which was closely related to haplotypes diM06, diM10, and diM11 that only occurred in the western Transverse and Santa Lucia Mountain ranges. The second cluster within this large group (diM05, diM14, diM18, and diM19) was found from localities in both the western and eastern portions of Transverse Ranges. The third cluster (diM16 and diM21) was found only in the eastern Transverse Ranges (San Gabriel and San Bernardino Mts.). The remaining haplotypes were unstructured and did not form distinct clusters from any particular geographic area.

In addition to higher haplotype diversity, *P. plicatus *mitochondrial haplotypes had more phylogenetic structure and geographic concordance, although few populations were resolved as monophyletic. The TCS haplotype network consisted of one large network and two disconnected singleton haplotypes (plM20 and plM31 from the northern Santa Lucia Mts and Santa Catalina Island, respectively) (Figure 3b). In the Bayesian gene tree, well supported clades distinguished haplotypes from the San Gabriel Mts. (plM22 - plM24 and plM27 - plM29), San Jacinto Mts. (plM09 - plM11), and Santa Cruz Island (plM05 and plM06) (Figure 2). One moderately well supported clade (pp = 0.87) joined haplotypes from the southern Santa Lucia Mts. (plM01 and plM18) with the one from Santa Catalina Island (plM31). Other clades with moderate support included some, but not all, haplotypes found in the Santa Ynez and Sierra Pelona ranges. Larger-scale patterns of relationship among regions were not evident.

The TCS network analysis based on the CAD data resulted in two networks corresponding to each of the two species. Haplotypes in the *P. diabolicus *network were closely related with few missing haplotypes and minimal geographic coherence (Figure 3c). Although not as highly divergent or exclusive as in the COI network, haplotypes in the southern areas were closely related, and exhibited high haplotype diversity relative to the number of individuals sampled (diN03-05, 11-13). However, this was not perfectly congruent with the COI results in several respects: 1) the Peninsular ranges exhibited no north-south split, 2) two (but not all) of the haplotypes from the San Gabriel Mts. were close to southern haplotypes, and 3) the Santa Monica Mts. haplotypes showed closer relationships to southern haplotypes than to northern ones. Diversity in the more northern areas (Santa Ynez Mts., northwestern Transverse Ranges, and Santa Lucia Mts.) was relatively low, especially considering the level of sampling in those areas.

On the other hand, CAD haplotypes in the *P. plicatus *network exhibited high diversity among individuals with many missing haplotypes, but were all connected in one network (Figure 3d). There was some geographic structure, as revealed by relatively close relationships of haplotypes from the northern and southern Santa Lucia Mts., Santa Ynez Mts., central Transverse Ranges, and the Santa Monica Mts. The eastern Transverse Range haplotypes (San Gabriel and San Jacinto Mts., as sampled for this species) were clustered in two separate groups; however, each had a surprising inclusion. One of these groups included all haplotypes from Santa Cruz Island (plN05-plN08), and the other contained one haplotype from the Santa Ynez Mts. (plN11). All other Santa Ynez Mts. haplotypes were in a lineage containing haplotypes from the northern and southern Santa Lucia Mts., central Transverse Ranges, and Santa Monica Mts.

In general, the results of the Φ_st _analysis illustrated that there was less structure among *P. diabolicus *populations than *P. plicatus *populations. However, significance of pairwise Φ_st _values varied for the two loci within each species (Tables 1 &2). For example, in *P. diabolicus*, the Φ_st _value between the geographically proximate Santa Ynez Mts. and northwest Transverse Ranges was significant in analyses of COI data, but not based on CAD. These discrepancies could result from the low number of CAD haplotypes in the *P. diabolicus *data set, especially within certain populations such as the northwest Transverse Ranges which only had two different haplotypes.

**Table 1:** *P. diabolicus *pairwise Φ_st _values by region.

	SStaLucia	SantaYnez	NWTrvRg	SanGab	SanBern	PeninRg
SStaLucia	-	0.321	0.122	0.076	0.481	*

SantaYnez	**0.345**	-	-0.004	0.219	0.360	*

NWTrvRg	-0.082	**0.323**	-	0.070	0.347	*

SanGab	**0.580**	0.040	**0.468**	-	0.136	*

SanBern	**0.190**	**0.318**	**0.354**	0.212	-	*

PeninRg	0.208	**0.316**	**0.432**	**0.208**	-0.183	-

Values for COI below diagonal, values for CAD above. Bold indicates significance at p < 0.05; asterisks represent cases where there was not enough data.

**Table 2:** *P. plicatus *pairwise Φ_st _values by region.

	SantaYnez	SanJac	SanGab	SanBern
SantaYnez	-			

SanJac	**0.463**	-		

SanGab	**0.636**	**0.828**	-	

SanBern	**0.214**	0.429	**0.714**	-

Values for COI below diagonal, values not calculated for CAD because of evidence for recombination. Bold indicates significance at p < 0.05.

The geographic structure differed in the two species based on the two-group AMOVA, as would be expected based on the phylogenetic and network analyses. In both species the highest percentage of variation based on the CAD gene came from high heterozygosity (within individual variation), regardless of group assignments (Table 3). In *P. diabolicus *within-population diversity greatly exceeded among-population or among-group diversity, regardless of which group populations were assigned to. In *P. plicatus*, the largest percentage of variation of the COI gene was among populations; however, AMOVA results for *P. plicatus *based on the CAD gene were dependent on how populations were assigned to the two groups. In sum, *P. diabolicus *exhibited less geographic structure than *P. plicatus *due to the fact that most of the genetic variation of *P. diabolicus *was represented within populations, while that of *P. plicatus *was mostly among populations (Table 3).

**Table 3:** Results of AMOVA analyses.

Species	Groups	Structure	Among gp. var. % (COI/CAD)	Among pop. var. % (COI/CAD)	Within pop. var. % (COI/CAD)	Within ind. var. % (CAD)
*P. diabolicus*	2	(S.SantaLucia)/(SantaYnez, NWTrvRg, SanGab, SanBern, [Penin])	-12.9/-9.4	32.1/23.7	80.7/33.5	52.0
*P. diabolicus*	2	(S.SantaLucia, SantaYnez)/(NWTrvRg, SanGab, SanBern, [Penin])	-7.0/0.6	32.2/18.8	75.0/31.5	49.0
*P. diabolicus*	2	(S.SantaLucia, SantaYnez, NWTrvRg)/(SanGab, SanBern, [Penin])	15.0/16.6	16.2/7.7	68.0/29.6	46.0
*P. diabolicus*	2	(S.SantaLucia, SantaYnez, NWTrvRg, SanGab)/(SanBern, [Penin])	32.0/26.0	6.4/6.6	60.9/26.3	49.0
*P. plicatus*	2	(SantaYnez)/([SierraPelona]/SanGab/SanJac)	-11.0/29.5	67.0/-6.0	44.0/-0.6	77.0
*P. plicatus*	2	(SantaYnez, [SierraPelona])/(SanGab, SanJac)	12.5/*	46.0/*	41.4/*	*
*P. plicatus*	2	(SantaYnez, [SierraPelona]/SanGab)/(SanJac)	3.2/-15.4	54.9/27.1	41.9/-0.7	88.9

Group designations described in text; brackets indicate regions included in COI analyses only due to small sample sizes of CAD; asterisks denote no CAD counterpart due to lack of data for Sierra Pelona.

When north-south populations were assigned to one of two groups in succession, the two species exhibited different geographic breaks (Table 3). In *P. diabolicus*, both genes had the highest among group variation when the southern populations (San Bernardino and Peninsular Ranges) were separated from all other populations (F_ct _= 32% (COI) & 26% (CAD). In *P. plicatus*, the among group variation based on the COI gene was very low with the highest being 12.5% (Santa Ynez Mts./Sierra Pelona + San Gabriel Mts./San Jacinto Mts.); however, among group variation based on the CAD gene was highest (F_ct _= 30%) when the northern population from the Santa Ynez Mts. was separated from the more eastern and southerly regions (Sierra Pelona, San Gabriel Mts., and San Jacinto Mts.).

**Figure 4:**
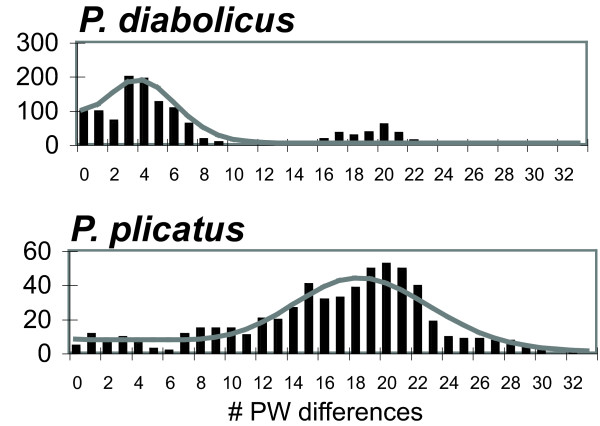
**Mismatch distribution based on the COI gene for *P. diabolicus *and *P. plicatus***. The *x*-axis represents number of uncorrected pairwise differences and the *y*-axis represents frequency.

The mismatch distributions revealed distinct demographic histories for each species (Figure 4). While both distributions were unimodal (non-significant Harpending's raggedness index: *P. diabolicus*, p = 0.67 and *P. plicatus*, p = 0.85), the mode for *P. diabolicus *was approximately four pairwise differences while the mode for *P. plicatus *was approximately 20 pairwise differences. This difference was reflected in the estimates of τ, which showed that the time of expansion for *P. diabolicus *(τ = 4.7; 95% confidence interval 1.8 - 7.2) was much more recent than for *P. plicatus *(τ = 19.1; 95% confidence interval 13.4 - 23.0).

## Discussion

Phylogeographic investigation of *P. diabolicus *and *P. plicatus *allowed us to directly compare the evolutionary history of each species and identify the extent to which each was affected by geography. Overall, *P. plicatus *haplotypes reflected a deep history with the majority of structuring occurring at the population level, while *P. diabolicus *exhibited little to no population structuring and low genetic diversity. This result supports a longer history for *P. plicatus *in this region, suggesting the current sympatric distribution is a result of recent movement of *P. diabolicus *into the area. The lack of concordant genetic breaks based on the AMOVA provides additional support for this hypothesis. In *P. diabolicus*, there was a prominent genetic break between northern and southern groups of populations, while *P. plicatus *showed most of the variation to be independent of larger geographic groupings. Thus, it appears that *P. diabolicus *populations have historically been isolated or restricted to the south. It is not clear whether these historical differences are due to geography, competitive interactions between the two species, or other factors not accounted for here. What is clear is that no single biogeographic barrier has had parallel effects on these two species.

If we use two proposed calibrations for insect mtDNA, 2.3% per million years [12] and 3.54% per million years [13], we can calculate an approximate range estimating the age of the most recent common ancestor of *P. diabolicus *and *P. plicatus*, in addition to the time to the most recent common ancestor within each species. The maximum uncorrected pairwise divergence between *P. diabolicus *and *P. plicatus *was 17.6% which would put the most recent common ancestor of these two lineages between 7.7 and 5.0 million years ago. The maximum pairwise divergence within *P. diabolicus *was 3.6%, and within *P. plicatus *was 4.1%. Thus, the most recent common ancestor of *P. diabolicus *haplotypes is estimated to have existed between 1.6 and 1.0 million years ago, relative to between 1.8 and 1.2 million years ago for *P. plicatus*. These estimates point out that, although the genetic patterns of these two lineages are quite different, the age of the most recent common ancestor of each is not.

Based on the mismatch distributions, it is apparent the time of expansion of *P. plicatus *preceded that of *P. diabolicus*. Thus, although our estimate for the most recent common ancestor of each these lineages is comparable, comparative analysis of these sister species provides an opportunity to contrast current diversity patterns with respect to demographic events that occurred at two different times. These results provide further support for the hypothesis that *P. plicatus *has inhabited this area for a relatively long period of time following a historical population expansion, with little to no current gene flow among populations. On the other hand, *P. diabolicus *appears to have undergone recent population expansion with high mobility throughout the sampled range.

There have been several other studies comparing the phylogeographic patterns of sympatric species in an attempt to isolate the factors affecting extant diversity patterns. In a study of sister tidewater goby species, Dawson et al. [4] examined the effects of differential dispersal abilities on phylogeographic structure. Similar to the results of our study, they found that one species was less structured with higher gene flow relative to the sister species. In the gobies, this difference was explained by species-specific traits related to habitat preference (correlated with opportunities for dispersal) and other natural history traits likely to affect gene flow. Steele et al. [3] compared the phylogeographic structure of two closely related sympatric salamanders and also found that life-history characters related to dispersal ability were largely responsible for the discordant phylogeographic structure in these species. In *Phloeodes*, variation in dispersal ability seems inadequate to explain differential phylogeographic structuring because 1) both species are flightless and there is no reason to expect unequal dispersal abilities, and 2) based on our collection records, these two species have largely overlapping habitat requirements precluding species-specific movement into many habitat types. Studies of such closely related species highlight how differential demographic histories might affect inferences from comparative phylogeographic studies on more distantly related taxa with unknown temporal dimensions.

It is evident that we're seeing only a small, relatively recent picture of each species' evolutionary history. This is evident by the long branches separating the species in both genes. This is not too surprising since our sampling is concentrated in the southern portion of both species' ranges. However, in temperate areas, it is often the case that southern populations source northern populations during times of post-glacial expansion, and on that basis we might have expected to see relatively ancestral populations for both species. But, as has been revealed in other studies, there have probably been more glacial refugia scattered around California, and perhaps in cooler and moister areas than in warmer, drier ones that are found, at least currently, in southernmost California [14-16]. The differing phylogeographic patterns shown for the two species may be due, in part, to having maintained substantial populations in different refugia through climatic fluctuations. It would be extremely valuable to expand the sampling in the future to cover these species entire ranges more thoroughly. This would undoubtedly reveal a more complete history of each, and allow a more definitive assessment of the factors associated with their divergence and their surprisingly different phylogeographic patterns.

## Conclusions

Based on two independently evolving genetic loci, this study revealed different evolutionary histories for the sympatric sister species, *Phloeodes diabolicus *and *P. plicatus*, in the beetle family Zopheridae. While the genetic data revealed unstructured populations in *P. diabolicus*, with evidence for a recent population expansion, *P. plicatus *exhibited geographically structured haplotypes with evidence of a more ancient population expansion. These data suggest that historical demographics have had a larger effect than geography on the extant diversity patterns of these two species.

## Methods

Taxon sampling was focused in the Transverse Range region but also extended into surrounding areas to the north in the Santa Lucia Mts. and south into the Peninsular Ranges (Figure 1). Specimens were collected under decaying logs and underneath the bark of dead trees. Genomic DNA was extracted from forebodies (head and prothorax) and legs of field collected specimens using DNeasy Tissue extraction kits (Qiagen, Valencia, CA). Apart from dissection, extractions were minimally destructive, and chitinous parts were mounted as vouchers following extraction. Full collection data, including voucher numbers and corresponding DNA extractions, can be accessed through the California Beetle Project database at http://www.sbnature.org/calbeetles. The latter half of the *cytochrome oxidase *subunit I (COI) gene was amplified using primers C1-J-1859 and C1-N-3014 [17]. We also amplified a portion of the nuclear *rudimentary *gene (CAD) using species-specific primers developed from an amplicon generated using the primers CD439F and CD688R [18]. Subsequent amplification was optimized by reducing degeneracy of the original primers, and shortening CD688R so that it began 13 base pairs downstream (CD439FPhlo 5' TTC AGT GTA CAG TTT CAT CCY GAG CAY AC 3' and CD688RPhlo 5' GGA TCG ACG TTT TCC ATG TTG CA 3'). PCR products were purified using QIAquick PCR Purification kits (Qiagen, Valencia, CA) or ExoSAP-IT^® ^(USB corp., Cleveland, OH). Forward and reverse sequencing reactions and sequence visualization was done by Macrogen, Inc. (Seoul, Korea). All DNA sequences were edited in Geneious Pro 4.5.4 (Biomatters Ltd., Auckland, NZ) and aligned in Se-Al v2.0a11[19]. Gene sequences were aligned manually and did not contain any indels. Recombination in the nuclear gene was assessed using the PHI (pairwise homoplasy index) statistic [20] implemented in the program SplitsTree v4.10 [21,22]. To test for patterns of selection at either locus, Tajima's *D *[23] was estimated in Arlequin v3.1 [24]. Significance was evaluated by comparing the observed test statistic to a distribution generated from 1000 permutations of the original data such that *p *values represent the proportion of the distribution that is less than or equal to the observed value.

### Data Analysis

Phylogenetic analysis of each gene was done in MrBayes v3.1 [25] to infer relationships among haplotypes. Models of molecular evolution for each marker were evaluated in MrModelTest v2.3 [26]. According to the Akaike Information Criterion, the COI data best fit the GTR + I + G model, and the CAD data best fit the GTR + I model. Gene trees were inferred in MrBayes, imposing the model specified by the AIC, using default priors. All haplotypes of both species were analyzed together for all phylogenetic analyses. The mitochondrial tree was rooted using the sequences of two other beetles in the family Zopheridae, *Zopherus granicollis *Horn. and *Phellopsis porcata *(LeConte) (sequences newly generated). Maximum uncorrected pairwise distances for each gene were calculated in PAUP* v4.0 [27]. We also constructed haplotype networks for each gene using TCS v1.21 [28] to obtain a non-bifurcating perspective of relationships. We used the default settings of a 95% connection limit.

In order to identify limits to gene flow and population structure across the sampling region, we computed pairwise Φ_st _values [29] among groups of populations, and performed Analyses of Molecular Variance (AMOVA), both using Arlequin v.3.1 [24]. Tested groups corresponded to major geographic regions (Figure 1) [16,30-32]. The goal of the AMOVA was to identify the deepest genetic division in each gene. Two-group analyses successively grouped different combinations of northern vs. southern regions together to test all possible single break scenarios (with the exception of the southernmost due to very small sample size for the single small southern population that would constitute one group; see Table 3 for all AMOVA comparisons).

A mismatch distribution for each of the species was generated in Arlequin [33] using the COI data only due to evidence for recombination of the CAD gene in *P. plicatus*. This distribution provides a graphic representation of the frequency of pairwise differences within a population, and can be used to estimate demographic parameters [34]. Here we compared estimates of the relative time of expansion (parameter τ), which allowed us to assess the relative time frame of population expansion for each species.

## Authors' contributions

MP carried out molecular genetic studies, data analysis, participated in study design, and drafted the manuscript. MSC conceived of the study, participated in its design and coordination and helped draft and revise the manuscript. All authors read and approved the final manuscript.

## Authors' Information

MP recently completed her postdoctoral research at the Santa Barbara Museum of Natural History. MSC is curator of the entomology collections at the Santa Barbara Museum of Natural History and is principal investigator of the California Beetle Project under which this research was conducted.

## Supplementary Material

Additional file 1 **Haplotype designations for COI and CAD with corresponding locality data, project voucher number, and GenBank accession number**. Click here for file
